# Druggable Sterol Metabolizing Enzymes in Infectious Diseases: Cell Targets to Therapeutic Leads

**DOI:** 10.3390/biom14030249

**Published:** 2024-02-20

**Authors:** W. David Nes, Minu Chaudhuri, David J. Leaver

**Affiliations:** 1 Department of Chemistry and Biochemistry, Texas Tech University, Lubbock, TX 79409, USA; 2 Department of Microbiology, Immunology, and Physiology, Meharry Medical College, Nashville, TN 37208, USA; mchaudhuri@mmc.edu; 3 School of Dentistry and Medical Sciences, Charles Sturt University, Wagga Wagga, NSW 2678, Australia; 4 Gulbali Institute for Agriculture, Water and Environment, Charles Sturt University, Wagga Wagga, NSW 2678, Australia

**Keywords:** ergosterol-dependent diseases, trypanosomes, fungi, irreversible enzyme inhibitors, sterol methyltransferase, CYP51

## Abstract

Sterol biosynthesis via the mevalonate-isoprenoid pathway produces ergosterol (24β-methyl cholesta-5,7-dienol) necessary for growth in a wide-range of eukaryotic pathogenic organisms in eukaryotes, including the fungi, trypanosomes and amoebae, while their animal hosts synthesize a structurally less complicated product—cholesterol (cholest-5-enol). Because phyla-specific differences in sterol metabolizing enzyme architecture governs the binding and reaction properties of substrates and inhibitors while the order of sterol metabolizing enzymes involved in steroidogenesis determine the positioning of crucial chokepoint enzymes in the biosynthetic pathway, the selectivity and effectiveness of rationally designed ergosterol biosynthesis inhibitors toward ergosterol-dependent infectious diseases varies greatly. Recent research has revealed an evolving toolbox of mechanistically distinct tight-binding inhibitors against two crucial methylation-demethylation biocatalysts—the C24 sterol methyl transferase (absent from humans) and the C14-sterol demethylase (present generally in humans and their eukaryotic pathogens). Importantly for rational drug design and development, the activities of these enzymes can be selectively blocked in ergosterol biosynthesis causing loss of ergosterol and cell killing without harm to the host organism. Here, we examine recent advances in our understanding of sterol biosynthesis and the reaction differences in catalysis for sterol methylation-demethylation enzymes across kingdoms. In addition, the novelties and nuances of structure-guided or mechanism-based approaches based on crystallographic mappings and substrate specificities of the relevant enzyme are contrasted to conventional phenotypic screening of small molecules as an approach to develop new and more effective pharmacological leads.

## 1. Introduction

**Figure 1:**
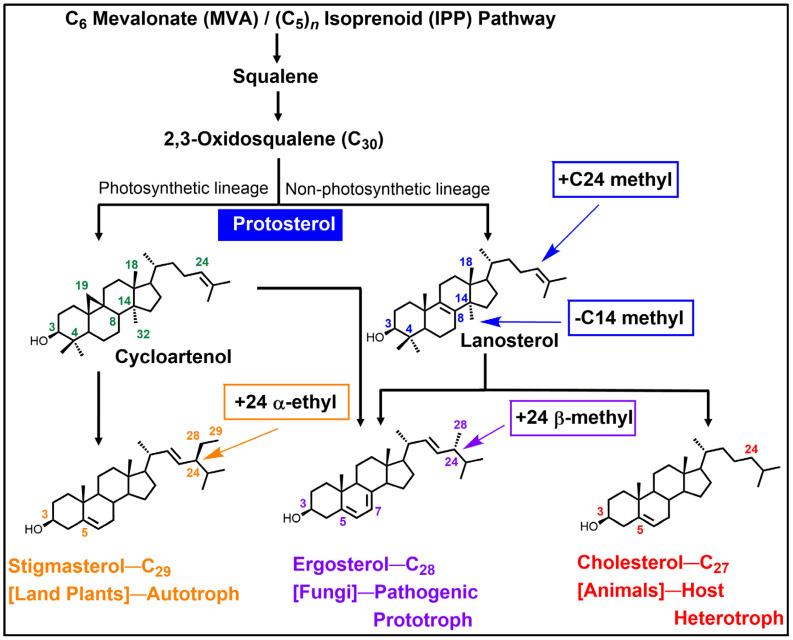
Cycloartenol-lanosterol bifurcation to alternate phyla-specific Δ^5^-sterol products synthesized in photosynthetic (auxotroph) and non-photosynthetic (prototroph/heterotroph) organisms. Note: key sterol molecular changes occur functionally with C14-demethylation and C24-methylation [1,2,3,4,5,6].

In this review, we are interested in crucial steroidogenic enzymes that act on sterol molecules [1,2] to produce membrane inserts—ergosterol (eukaryotic pathogenic organisms: fungi, trypanosomes and amoebae) and cholesterol (animal hosts) [3,4,5], and the medical relevance in targeting active site structures and reaction mechanisms of sterolic enzymes notably those sensitive to substrate analogs that can bind irreversibly to combat ergosterol-dependent diseases (Table 1). More specifically, this review highlights the award lecture for the sterol prize-George Schroepfer Medal presented to W.D.N. at the 2019 AOCS meeting in Saint Louis (United States), where the enzymology and inhibition of two universal enzymes—the C24-sterol methyltransferase (C24-SMT) and C14-sterol demethylase (C14-SDM; CYP51p), involved in the addition or removal of a methyl group from the protosterol molecule in ergosterol-dependent diseases was discussed (Figure 1). Steroidal addition-elimination reactions in primary metabolism have long been understood and recognized to proceed in a phyla-specific manner [6]. Current indications suggest they are not genetically or mechanistically similar nor catalytically reversible under physiological conditions. Nor is there a specified conserved region in the amino acid sequences of these enzymes for a common sterol binding site [7]. These facts, not withstanding recent distinctions in the chemical biology of the associated enzymes-C24-SMT and C14-SDM, have been unearthed making for renewed study of them in a range of pathogenic organisms that synthesize ergosterol—a topic of intense research [8,9,10,11,12,13].

## 2. For Context: Background for C24-SMT and C14-SDM Enzymes in Sterol Biosynthesis Pathways

**Figure 2:**
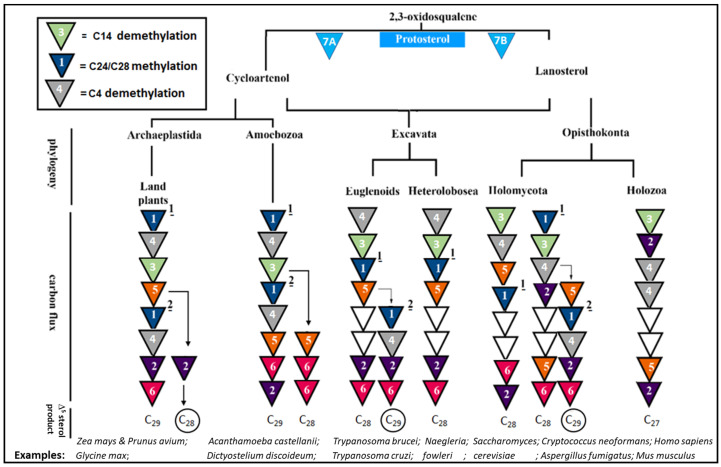
Phylogenetic distribution of representative pathogenic trypanosomes, amoeba and fungi and their animal host synthesizing Δ^5^-sterols [1,2,3,4,5]. Triangles (T) including numbers indicate distinct biosynthetic steps; C_27_ = H at C24, C_28_ = CH_3_ at C24, and C_29_ = C_2_H_5_ at C24. T1 = sterol 24 methylation (^1^ implied first C_1_-transfer at C24; ^2^ implied second C_1_-transfer at C28), T2 = sterol C24(25)- or C24(28)-reduction, T3 = C14-demethylation and C14-reduction, T4 = C4 demethylation and C3-reduction, T5 = the typical sequence of 9,19 cyclopropyl ring opening to Δ^8^ followed by Δ^8^ isomerization to Δ^7^ followed by Δ^5^ desaturation followed by Δ^7^ reduction yielding the Δ^5^-monene product. T6 = Δ^22^ desaturation, T7A and T7B = occurs first in the sterol metabolism sequence to create the protosterol which involves one or the other synthase (cyclase) to produce cycloartenol or lanosterol. Circle around C_28_ and C_29_ sterol compounds represent products of parallel sterol methyltransferase pathways generated from a common branchpoint and can involve genetically distinct sterol methyltransferase enzymes identified as SMT1 and SMT2, isoforms with different substrate preferences (Table 2). Adapted from [1,4,6,24].

From an evolutionary perspective, steroidogenesis proceeds through a sterol structure-determining step to produce the so-called protosterol, a C_30_-molecule that originates biosynthetically from the cyclization fate of 2,3-oxidosqualene. As often happens in protosterol formation, a lanosterol-cycloartenol bifurcation can arise first in the sterol biosynthesis pathway from which phylogenetic differences in the enzymes constituting these variant pathways yield Δ^5^-sterol products in one of the major eukaryote kingdoms of plant, animal of fungi (Figure 2 and Table 2) [4,6]. In photosynthetic plants, in non-photosynthetic pathogenic amoebae (*Acanthamoeba* sp. and *Naegleria* sp.) and select non-photosynthetic pathogenic algae (*Prototheca* sp.), cycloartenol synthase converts 2,3-oxidosqualene into cycloartenol [14,23,24,25,26], while in fungi, animals, kinetoplasts the lanosterol synthase generally converts 2,3-oxidosqualene into lanosterol [2,15,27]. As shown in Figure 2, these protosterols are further converted to Δ^5^-sterol products with distinct canonical patterns resulting from a kinetically favored pathway that is regulated by individual enzyme substrate specificities and their corresponding turnover rates in the Archaeplastida and Amoebozoa versus the Excavata and Opisthokonta. By following the triangles 1 and 3 in the biosynthetic pathways outlined in Figure 2, it is evident that the enzymatic order for sterol the C24-methylation-C14-demethylation reactions can switch position depending on the taxonomic group of enzyme origin while both protosterol is capable of conversion to ergosterol (Figure 2). For example, in the pathogenic amoebae *Acanthamoeba castellani* that synthesize cycloartenol and ergosterol, the pathway proceeds with sequential C24 methylation, C4 demethylation and C14-demethylation [14]. Alternatively, in pathogenic protozoa that synthesize lanosterol and ergosterol, as in the euglenoid *Trypanosoma brucei* [28], C4-demethylation proceeds first followed by C14-demethylation, then C24-methylation. This variation in enzyme order can extend into the animal lineage (minus C24 SMT participation) as noticed in cholesterol biosynthesis from lanosterol in cancerous and non-cancerous tissues ([2] and references cited therein).

Most pathogenic fungi, exemplified by the basidiomycetes *Cryptococcus* which synthesize ergosterol [21] possess a hybrid of the previously discussed pathways where C24-methylation biosynthetically occurs first to modify the protosterol followed by C14-demethylation, then C4-demethylation. This biosynthetic sequence for C24-methylation followed by nuclear demethylations that include the pathogen *Candida albicans* [18], contrasted with the ergosterol biosynthetic pathway defined in the ascomycetes non-pathogenic yeast-*Saccharomyces cerevisiae* where C14-demethylation occurs first followed by C4-demethylation (to yield cholesta-5,24-dienol or zymosterol), then C24-methylation [7]. Interestingly, the different routes to the same end-product can introduce to the sterol core a C24-alkyl group or removal of a C14-methyl group—necessary features for membrane congruence of ergosterol [29]. Although not generally considered, it would appear the positioning of C24-SMT and C14-SDM in ergosterol formation could be a functional determinant in ergosterol depletion. As such, the endogenous levels and timing of expression of the individual C24-SMT and C14-SDM may contribute to whether inhibitor treatment of the paired enzymes leads to growth static or cidal affects in ergosterol-dependent organisms.

### 2.1. Substrates in Ergosterol Biosynthesis

Not only are the structural features of sterol side chain methylation and nucleus demethylation relevant to the ergosterol-cholesterol function in membranes, but they are also now recognized in the specificities of substrate and inhibitor binding amongst the different families of sterol methylases and demethylases. To this end, in the later part of the 20th Century, the identity of favored substrates for some C24-SMTs and C14-SDM in non-pathogenic fungi and plants were determined and compared to their animal counterpart through structure-activity studies using crude microsomal preparations [30,31,32,33,34]. These early in vitro studies failed to show clear differences in animal and fungal CYP51 substrate preferences while no comparison could be made for fungal/plant C24-SMT against human C24-SMT since the latter does not exist in the human proteome. The extensive natural product studies determined from 1965 to 1985 were often interpreted to show crisscrossing sterol biosynthesis pathways in for example, yeast and corn, as a result the endogenous sterolic enzymes were assumed to generally lack substrate specificity.

**Figure 3:**
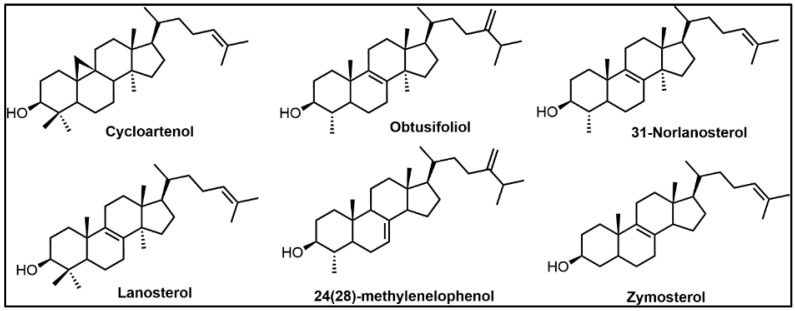
Typical preferred substrates for C24-SMT and C14-SDM enzymes represented in biosynthetic steps in triangle 1 or 3 in Figure 2, as discussed in the text.

In more recent sterol identification and ^13^C/^2^H-labeling biosynthetic studies coupled with investigations of cell-free enzymes—a high degree of substrate preference for C24-SMT or C14-SDM was observed and correlated to kinetically favored canonical sterol biosynthesis pathways that are now known to possess a specific number of genes corresponding to each sterol enzyme [25,31,33,34] (cf., Figure 2). For example, cycloartenol is shown preferred by SMT1 in plants while 24(28)-methylene lophenol is preferred for SMT2 in a range of plants and non-photosynthetic organisms while lanosterol and zymosterol are the substrate of choice in many non-photosynthetic organisms (Figure 3). Alternatively, lanosterol or 31-norlanosterol can be the preferred substrate for C14-SDM in non-photosynthetic organisms, while 24(28)-methylene lophenol or obtusifoliol can be the preferred substrate for C14-SDM in plants as well as non-photosynthetic organisms (Figure 3). Collating the in vitro data obtained by Michaelis Menten kinetics (*K_m_* and *V_max_*) against favored substrates for cell-free enzymes representing the fungal ergosterol (*S. cerevisiae*) and plant sitosterol (*Zea mays*) biosynthesis pathway (Table 2) [30,31] enabled the rate-limiting biosynthetic step to be inferred. These studies showed that for representative organisms, C4-demethylation is the slow acting step in the fungus while C24-methylation is the slow acting step in the plant, verified independently by comparing isolated enzyme activities with total sterol content and labeling studies performed during plant and fungal growth [27,34,35,36,37]. In rats and humans where sterol C24-methylation does not exist, sterol C14-demethylation is rather fast as it is in fungi, but much slower in protozoa by a factor of 5-6 [14,33,38]. Taking note of these findings and first generation testing of azoles against C14-SDMs or transition state analogs against C24-SMTs [39,40] provided a growing awareness in the late 1980s and 1990s for: (i) a uniformity in sterol biosynthesis in many fungi and that these ergosterol biosynthesis pathways differed from the sterol biosynthesis pathways operating in plants and animals, and (ii) an appreciation for the vulnerability in sterol methylation-demethylation reactions involved with ergosterol-dependent diseases so that unique ergosterol biosynthesis inhibitors could be developed that would not harm the animal host.

Intriguingly, research into the development of new and effective therapeutic leads against ergosterol-dependent diseases using enzyme-based approaches was stalled during the 1980s, for sterolic enzymes were membrane-bound and notoriously difficult to purify to homogeneity for mechanistic or kinetic studies. However, with the introduction of improved molecular biology tools in the 1990s investigators afforded programs into the cloning and overexpressing of these enzymes, which then led to the first complete purification of a C24-SMT which in turn led to the first determinations of amino acid sequence relatedness among this class of enzyme. Additionally, determination of turnover numbers (*k_cat_*), requiring knowledge of the C24-SMT/C14-SDM amounts in enzyme preparation and detailed characterization of the reaction mechanisms [41] were made possible with the availability of cloned sterolic enzymes [41,42].

### 2.2. The Evolution of Antifungal Drugs Targeting C24-SMT and C14-SDM in Ergosterol Biosynthesis

The advent of antifungal/antibacterial screening of natural products from fungi and bacteria led to the discovery of penicillin for the treatment of bacterial infections in the mid-20th Century, well before any significant thought was directed at the inhibition of ergosterol production and processing in eukaryotes. With successes in antibacterial treatment using penicillin, focus then switched to the possibility of identifying fungal antibiotics which then led to the discovery of Amphotericin B (AmB) that was found to be a potent antifungal drug (Table 3) [43]. The mechanism of action of AmB is to complex with membrane ergosterol—inducing membrane leakage and rapid fungal cell death [44], suggesting for the first time an ergosterol-dependency in pathogens. Because of its effectiveness, AmB has become the gold standard for antifungals over the last 50 years. Interestingly, the mode of inhibition of penicillin is distinct from AmB which is a suicide inhibitor that interferes with cell wall biosynthesis uniquely in the prokaryote domain [45]. As evident in Table 3 and as new chemistry areas evolved—new therapeutics were recognized as antifungals. An important new group of compounds spotted from phenotypic screening methods in the 1960s, the “azoles”- a class of five-membered heterocyclic compounds containing a nitrogen atom and at least one other heteroatom, were discovered serendipitously. The mechanism of inhibitor action for these chemically simple growth inhibitors came later after adequate sterol analytics evolved in the 1970s to screen total lipids extracted from fungi. Ultimately, the site of inhibitor action was shown to be within the ergosterol biosynthesis pathway, and at the C14-demethylation step catalyzed by C14-SDM (CYP51p). Depending on the fungus examined, this biosynthetic blockage typically resulted in an accumulation of lanosterol, its C24-methyl analog: 24(28)-methylene dihydrolanosterol (eburicol) or one of the C4-demethylated lanosterol derivatives ([46] and references cited therein).

Imidazole type drugs, such as clotrimazole, miconazole, and ketoconazole are first generation azoles prepared initially for clinical testing, followed by multiple generations of triazoles used in medicine and agriculture [47], that include fluconazole, itraconazole, voriconazole, posaconazole, isavuconazole—all of these drugs have successively entered the clinic. Interestingly, early use of miconazole in animal cell-based cultures produced a biphasic modulation of HMG-reductase (rate-limiting enzyme in cholesterol biosynthesis) which correlated well with the buildup of the lanosterol metabolite, 3β-hydroxylanost-8-en-32-al—an oxysterol regulator of cholesterol production [48,49] that was generated during treatment.

Several second generation CYP51 inhibitors evolved in the treatment of topical or superficial fungal infections, such as oxiconazole, sertaconazole, luliconazole, efinaconazole, and ravaconazole. Chemically, the N3 of the imidazole compound is shown to bind to the heme iron atom of ferric cytochrome P450, whereas the N4 of the triazoles binds to the heme group on the sterol demethylase, binding parameters used in C14-SDM inhibition studies [19,50]. Selective toxicity against pathogenic fungi of azole drugs is believed to derive from the greater affinity of azole for the fungal CYP51 compared to the human CYP51, for which there is supporting data [19]. With further development, these novel azoles were shown to have wide applications in medicine to treat for example, ringworm, topical yeast infections, aspergillosis, candidiasis, and nail fungus onychomycosis (Table 3), as well as for crop protection [46,51]. Many of them have been commercialized after initial phenotypic screening coupled to minimum inhibitor concentration curves of inhibitor yielding cell death in the low micromolar range [46,51]. Still, there has been much caution recently in treating infections with azoles because both the imidazoles and triazoles can produce resistance and/or produce adverse reactions caused by inhibition of related human cytochrome P450-enzymes due to their strong affinity toward heme iron [52]. However, replacement by 1-tetrazole in the azole structure has shown promise to attenuate such physiological and biochemical issues. Indeed, new compounds VT-1611 (oteseconazole), VT-1129 (quilseconazole), and VT-1598 have appeared [53] and are shown to possess the desired fungal C14-SDM specificity for binding properties that favor the fungal CYP51 over the human CYP51 [19].

**Table 3:** Evolution of medically relevant antifungal drugs targeting ergosterol dependency in fungi ^1^.

Mechanism of Action		Block C14-Demethylation	Block C24-Methylation		Complex with C_28_-Sterol
**Biosynthesis Pathway**	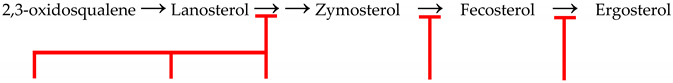				
**Time** **Introduced**	Early 1980s	Early 1990s	2022	2007	Early 1960s
**Drug Class**	Imidazoles 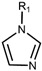	Triazoles 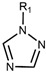	Tetrazoles 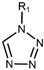	Arylguanidines 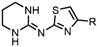	Polyene Antibiotics 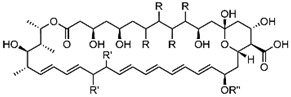
**Drug** **Examples**	Clotrimazole Ketoconazole Miconazole	Itraconazole Posaconazole Efinaconazole Fluconazole	Oteseconazole	Abafungin	Amphotericin B Nystatin
**Drug/Use/Examples**	Canestin/Ring Worm and Topical Yeast Infections Nizoral/Anti-dandruff Shampoos Monistat/Topical Yeast Infections	Sporanox/Ring Worm and Candidiasis Noxafil/Prophylaxis for Aspergillosis and Candidiasis Jublia/Nail Fungus-Onychomycosis	Vivjoa/vaginal Yeast Infections/ Candidiasis	Abasol/ Nail Fungus- Onychomycosis/Dermatomycoses	Fungizone or Amphocin/Systemic Fungal Infections/Aspergillosis and Candidiasis Fungicidin or Nystatin Cream/Topical Yeast Infections/Candidiasis

^1^ Adapted from [39,40,43,44,45,46,52,54].

Since 1978, the C24-methyl group in the ergosterol side chain introduced through the actions of C24-SMT has been understood to be functionally essential in yeast growth [29]. More recent study studies of the C24-SMT gene-ERG6 involving ERG6 deletion mutants in various pathogens shown to be more susceptible to azoles have confirmed the importance of C24-SMT activity in fungal and protozoa physiology [55,56] and paved the way for the commercialization of a C24-SMT inhibitor drug (abafungin) in early 2000. Abafungin possesses a cyclic guanidine moiety (Table 3) and was identified fortuitously as a C24-sterol methylation inhibitor [54]. However, there is no mechanistic basis for an aryl guanidine-type inhibitor to interfere with C24-SMT activity in ergosterol biosynthesis. Like azoles, this compound is not sterol-based. Abafungin was discovered through phenotypic screening methods and was reported to block sterol C24-methylation of zymosterol [54], the native substrate of Erg6p in *S. cerevisiae*. The drug has been used to treat nail fungus onychomycosis (Table 3). Further, abafungin was observed to have MIC values of 0.5–16 mg/mL and 0.5–1 mg/mL against *C. albicans* and *A. fumigatus*, respectively [54]. Abafungin exhibited excellent minimum fungicidal concentrations of 20–80 mg/mL, 10–80 mg/mL, 1.3–2.5 mg/mL against *C. neoformans*, *C. albicans* and *A. fumigatus*, respectively [54]. To date, no other C24-SMT inhibitor has been commercialized as an antifungal likely for mechanistic reasons related to similarities in HEI formation during catalysis of C24-SMT and C24-sterol reductase [57]. Experimental support for similarities in specific enzyme reaction cycles in ergosterol and cholesterol biosynthesis that can be compromising to drug development was noted in enzymic studies of transition state analogs targeting C24-SMT in fungi and C24-sterol reductase activity in animals inhibited both enzymes equally and with high potency [58,59].

### 2.3. C24-SMT: Differences in Catalytic Competence and Product Distributions in Non-Pathogenic and Pathogenic Organisms

Sterol C24-methylation is an essential part of ergosterol biosynthetic pathways in non-pathogenic (e.g., *S. cerevisiae*) and pathogenic fungi (*C. albicans* and *C. neoformans*) providing the 24β-methyl group in the ergosterol side chain. The C24-SMT enzyme catalyzing this reaction was first cloned and heterologously expressed in *Escherichia coli* and purified to true homogeneity in 1998 [41]. In the years that followed, orthologous plants and protozoa SMTs were cloned, expressed, and purified in similar fashion to Erg6p. With <10% of residues in SMT identical across kingdoms for plants, protozoa and fungi—the catalytic mechanism is reportedly the same, and involves a methylation-deprotonation reaction subject to a common sterol substrate having a C3-hydroxyl group, planar nucleus, and intact Δ^24^-bond in the sterol side chain [30,32,37]. However, the transition state coordinate in the sterol methylation reaction cycle, studied using a series of chemically modified transition state analogs against various fungal and plants enzymes, can differ according to phylogenetics [30,58,59].

**Figure 4:**
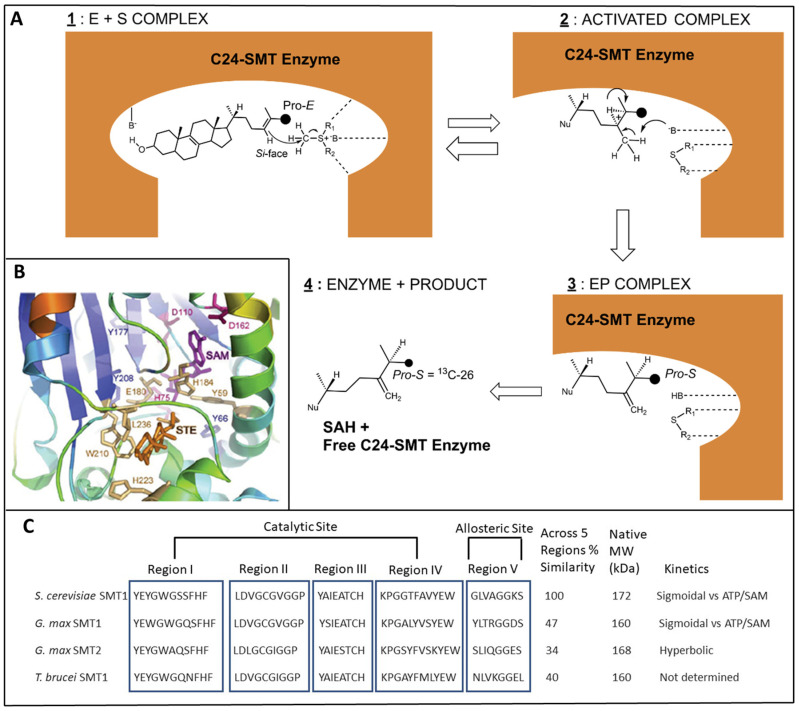
Representative C24-SMT enzyme properties. (**A**) Steric-electric plug model for C24-SMT catalysis showing putative active site contribution to sterol methylation, adapted from [37,60]; C24-SMT = 24C-sterol methyltransferase enzyme, B = unspecified base, E = enzyme, P = product, SAH = S-adenosyl-L-homocysteine. The reaction mechanism predicts Si-face (backside) or β-methyl attack on the Δ^24^-double bond with the terminal ^13^C26 Pro-E methyl group converted during the reaction course to the C26 Pro-S methyl (c.f. Ref [29] for a deeper discussion of sterol structure and nomenclature). Note: when C27 is labeled with ^13^C the ProRC27 on the Δ^24(25)^-double bond undergoing sterol methylation at C24 yields a C25(27) ProR configuration in the product [63,64,65,66]. In this study we use the biosynthetic side chain rule [63], which recognizes C26 and C27 as originating in C2-and C6-MVA, respectively, while the IUPAC nomenclature fails to recognize the chemically equivalent C26 and C27 atoms as biochemically or magnetically different. (**B**) Ribbon representation of the active site of TB C24-SMT [74], adapted from the homology model presented first for *S. cerevisiae* C24-SMT [75]. The SAM (*S*-Adenosyl methionine);magenta) and sterol (STE; orange) binding segments are shown. (**C**) Conserved SMT motifs that segregate into an active site (Regions I to IV) and an allosteric site (Region V) as determined using Clustal Omega for amino acid sequence relatedness, mutagenesis and affinity labeling ([74,75,76,77,78] and references cited therein).

The transition state coordinate in SMT catalysis is typically composed of a series of high energy intermediate cations (HEI) at C25 (HEI-1) and C24 (HEI-2), respectively, that are eliminated by a C28 deprotonation step to produce a C24(28)-methylene sterol, as envisaged in the steric-electric plug model for sterol methylation (Figure 4A) [37]. Sterol C24-methylation has been shown to proceed with a high degree of regio- and stereo-specificity in vivo [60,61,62,63] and in vitro [41,64,65,66,67,68,69,70,71] for both the first and second C24-methylations following incubations with ^13^C- and ^2^H-labeled substrates and using ^13^CNMR and ^1^HNMR for product identities [61,62,63,64,65,66,67,68,69,70,71]. The strict reactions are enabled by a flexible sterol side chain which can assume a productive conformation relative to the cofactor when bound to the C24-SMT enzyme (Figure 4).

**Figure 5:**
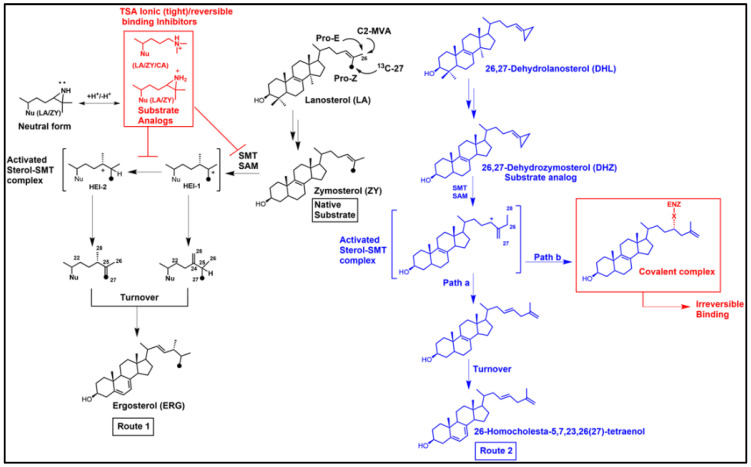
Representative sterol methylation routes to product turnover and inhibition. Methylation of a native substrate at C24 can produce a product on Route 1 [41] that converts to ergosterol while methylation of a substrate mimic at C26 can produce an alternate product on Route 2, Path a, that converts to an unnatural analog with a lengthened sterol side chain recognized as suitable for fungal growth [76,77,78]; or this analog can produce irreversible-type inhibition from covalent binding (ester bond) of the C24-intermediate ion to the enzyme, Path b (ENZ; X is an unspecified base in the active site) that can lead to depleted ergosterol and cell death. Nu= unspecified sterol nucleus, HEI-high energy intermediate. Dot in C27 is meant to show ^13^C (See Figure 4).

For some pathogenic amoeba (*A. castellanii*) [14,72] and kinetoplasts (*T. brucei*) [73], as well as pathogenic colorless-algae typified by the yeast-like alga-*Prototheca wickerhami* [69], substrate binding can produce an alternate product from the Erg6p product, that is a C24-methyl C25(27)-olefinic product that can be converted to ergosterol (Figure 5). For *A. castellanii*, a second sterol methylation of the C24(28)-sterol substrate can also occur, but in this case the biosynthetic step requires a separate C28-methyl transferase (SMT2), like in plants [71,72], and is responsible for the catalysis of the ∆^25(27)^-methylation reaction while AcSMT1 generates the C24(28)-methylene product. Alternatively, in *T. brucei* a single C24-SMT is responsible for the first C1-transfer reaction yielding multiple products of a ∆^25(27)^-methyl sterol product (major), and a ∆^24(25)^-methylation product (minor); the later product then can undergo a second C1-transfer reaction at C28 to produce a novel ∆^25(27)^-C24-dimethyl sterol [73].

As inferred in Figure 2, the C24-SMT enzyme is synthesized in all major groups across the eukaryote domain. Many C24-SMTs have been examined for sequence relatedness and found to possess a high degree of similarity (ca. 50%) [79]. The C24-SMT has an active site for sterol substrate and SAM cofactor (with a Rossman fold) which spreads across the protein structure as Regions I to IV [7,78], while a conserved Region V (with a Walker sequence) in several C24-SMTs have been unearthed as a second binding site for SAM in the native tetrameric structure [76,77]. Cooperativity among its subunits was first suggested by sigmoidal behavior from fixed zymosterol and varied SAM kinetic experiments against the yeast C24-SMT [40] and from ATP allosteric effects on affecting rates of sterol methylation [76,77]. Binding affinities for ATP, SAM, and zymosterol have been determined against the Erg6p and in each case the ligand generated *K_d_* values of approximately 4 μM [75,77,80].

Further work on the cloned enzyme clearly defined two saturation plateaus, one at 100 μM (typically the plateau studied by researchers investigating SMT action) and one at 400 μM [76,77]. The former kinetic studies yield a sterolic enzyme that can be a very slow-acting with *k_cat_* of 0.6 min^−1^ while it can undergo increased catalytic rates through allosteric regulation affording a maximum *k_cat_* of approximately 5 min^−1^ which is more in line with the lower end *k_cats_* of C14-SDMs across kingdoms [37]. These kinetic data support C24-SMT as a rate-limiting enzyme for some fungal, plant and amoeba steroidogenesis. When multiple copies of C24-SMT exist, and one of them contributes to the second methylation steps, it appears the C24(28)-SMT is not allosterically activated by SAM or ATP and can differ in their Region V amino acid composition from that of yeast C24-SMT [76,77] (Figure 4C). Another kinetic constant established for each of the representative enzymes reported in Table 4 is the activation energies (*E_a_*) to catalysis which can differ in a phyla-specific manner [78,80].

Although the enzyme has been found to be recalcitrant to crystallization, we succeeded first at purifying a cloned C24-SMT enzyme to homogeneity [41], then in generating the native protein for structure determination [81]. Our collaborator at the University of Pennsylvania showed Erg6p diffracts to about 4Å, which is not sufficient diffraction to determine the enzyme structure [82]. In the absence of a successful X-ray structure for C24-SMT, we attempted to generate a homology model of the active site cleft bound by sterol and SAM (Figure 4B; Ref. [74]) by the following criteria: (1) Mutagenesis experiments targeting conserved acidic residues considered important in deprotonation reactions [74]; (2) Identifying aromatic residues considered important in cation (charged C24-methyl intermediate)-π (aromatic residues that produce negative counterions) interactions that determine product specificity in C24(28)/C25(27)-olefins or in C24- and C28-alkylated sterol side chains [7,66,75,83,84,85,86]; (3) Generating a suicide inhibitor that upon complex to Erg6p binds to an active site acidic residue with an ester (covalent) link [85,86,87].

The properties of C24-SMTs for fungi and protozoa are similar in amino acid count ranging from AcSMT2 at 346 to ScSMT at 383 with isoelectric points differing from 6.40 to 5.54, respectively and the corresponding molecular weights for the monomeric form ranging from 39 kD to 43 kD, respectively (Table 4). In comparison to the ScSMT amino acid composition, the primary amino acid sequences of C24-SMT in the pathogenic fungi, trypanosomatids, amoebae and plants that we have studied, show the following similarities; CaSMT-51%, TcSMT-39%, NfSMT-39%, AcSMT1-43%, AcSMT2-43%, GmSMT1-47%, and GmSMT-35%. However, the genomic organization of the enzymes are not the same. Indeed, the plant SMT2s possess an intron organization of twelve while, for example the ScSMT and TbSMT is zero [74]—the significance of these genomic differences is not evident. Substrate preferences for representative C24-SMTs are distinct: (i) the *C. albicans* C24-SMT prefers zymosterol with lanosterol not productively bound to the enzyme [81], (ii) the amoeba *A. castellanii* C24-SMT (SMT1) prefers cycloartenol (C4-dimethyl sterol) but can bind zymosterol weakly while the SMT2 protein prefers obtusifoliol (C4-monomethyl sterol) with cycloartenol binding weakly [72], and (iii) the Kinetoplastid *T. brucei* prefers zymosterol (C4-desmethyl sterol) as substrate with lanosterol not productively bound to the enzyme [73] (Figure 3).

We have also considered the phylogenetic implications of the steric-electric plug model, relevant to the primary structures of a range of C24-SMT [7,24,68,78,79]. In summary the extant enzyme likely evolved from an ancestral C24-SMT through a process of gene duplication and divergence of key catalytic and allosteric residues that have influenced catalytic rates and product outcomes in a phyla-specific manner. The observed differences in product specificity and sensitivity to substrate analog inhibitors among C24-SMT enzymes likely arose through mutations which changed the shape of the methylating active sites and/or the position of the crucial functional groups involved with cation-π interactions and deprotonations that occur during catalysis. Possibly, in organisms that operate on the step-wise sterol methylation route yielding the C25(27)-sterol product is ancestral and preceded organisms that yield the C24(28)-product since the HEI on the path to C25(27)-olefins occurs first in the reaction cycle and in the activation energy to HEI-C24 cation formation is of higher energy compared to the energy barrier to form HEI to C25 cation, as noted by the *E_a_* constants for TbSMT and ScSMT catalysis against similar substrates (Table 4). Consequently, it may be that the observed phylogenetic differences in amoeba, *Trypanosoma*, and fungus C24-SMT activities could be a factor in the enzyme sensitivity to suicide inhibitor effectiveness in killing parasites, discussed next.

## 3. C24-SMT Sterol Biosynthesis Inhibitors

### 3.1. Inhibition of Ergosterol Biosynthesis Using Reversible-Type Inhibitors: The Transition State Analogs (TSA) Targeting C24-SMT

Transition state analog inhibitors designed to inhibit C24-SMT have been studied extensively for over 50 years. Kinetically, they have been shown to bind to the Erg6p with similar affinities as the zymosterol substrate, *K_d_* of 25-azalanosterol is approximately 4 μM [75] and to bind reversibly, albeit tightly, to soybean C24-SMTs [67]. As transition state analogs, the inhibitors are designed with the idea that the cationic positive charge from, for example, a nitrogen atom, positioned along the lateral sterol side chain is paramount for inhibitor activity while the sterol structure requirements noted for substrate binding, such as for a C3-OH and Δ^24^-bond [30,36,69,71,80] are less important or not at all. In our work, full understanding of sterol methylation reaction mechanisms requires elucidation of the structures of the transition states. Studies of stable analogs of the substrate portion of the activated complex (Figure 4A) can reveal what substrate bond-making-bond-breaking steps occur during turnover and which features of the molecule can compromise turnover leading to covalent attachment of the intermediate ion. The chemical mechanisms for C24-SMT catalysis, which includes the methyl cation from cofactor SAM and nucleophilic olefin from substrate sterol side chain, can generate a panoply of C24-methyl/ethyl products so long as the intermediate cation is not quenched prior to turnover. [60]. What appears to be characteristic of C24-SMT inhibition by transition state analogs is the tightness of binding that spans the range of 5 nM to 500 nM [30,59,68,72]. These inhibitors generally show non-competitive inhibition kinetics with respect to the natural substrate of C24-SMT. Using 25-azalanosterol as a representative TSA against C24-SMT, the binding constant (*K_d_* 4 μM) and approximate inhibitor constant (K*_ι_* 20 nM) show major differences against Erg6p for catalysis [75,80,85,87]. The data suggest the enzyme active site can assume an initial conformation appropriate for binding the ground state of the inhibitor analog while a second conformation ensues following binding that yields a readjustment in active site structure to accommodate and fine-tunes the tight binding of the TSA sterol side chain (with its protonated nitrogen atom yielding a positive charged sterol side chain similar in structure to the HEIs produced during sterol methylation) into an inhibitor-enzyme complex that effectively derails C24-SMT catalysis.

The rational design and testing of these compounds have a long history that date back into the 1980s where focus was on establishing the potential for inhibition of sterol C24-methylation as a mechanism to generate ergosterol-dependency in pathogenic fungi causing agriculture problems. Indeed, such TSA analogs, viz. 24(*R*,*S*)25-epiminolanosterol (EL) which originated in our laboratory, were found to be highly effective in disrupting mycelia growth [88]. However, when the same transition state analog inhibitor targeting the fungal C24-SMT, EL, was examined against a crop plant (sunflower) C24-SMT, the inhibitor was determined to be highly potent in vivo and in vitro exhibiting a *K_i_* against the microsomal enzyme in the nanomolar range [89], showing the compound, and this class of sterol biosynthesis inhibitor, was not a suitable antifungal candidate for crop protection. When EL was tested against the protozoan *Trypanosoma cruzi*, its potent antiproliferative effects and targeted inhibition of C24-methyltransferase were shown to cause an accumulation of zymosterol and loss of ergosterol with increased concentration to the growth medium which suggested it to be a possible lead therapeutic [90]. Subsequent research on EL and other transition state analogs against the cloned and purified TcSMT yielded *K_i_* constants in the predicted low nanomolar range, confirming TcSMT as a target for these compounds under physiological conditions [91,92]. In further study of TSAs in a mouse model of *T. brucei* infection, the inhibitors were reported to reduce the infection burden and extend the life of animals for several days, but the drugs were also found to force an accumulation of desmosterol relative to cholesterol synthesis in liver sterol analyses (Nes, unpublished), which again shows some compounds may not be useful therapeutic leads. In support of the liver analyses just described, we also reported that EL incubated with cultured rat hepatoma cells generated an accumulation of cholesta-5,7,24-trienols and loss of newly synthesized cholesterol, consistent with the treatment inducing blockage of the sterol C24-reductase enzyme [93]; these studies are also supported by those of Ator et al., showing in vitro testing of TSAs against microsomal preparations of rat sterol C24-reductase led to lost activity [58,59].

Another approach using TSAs is for sterol profiling of pathogenic organisms, for example, treated fungal (*Candida*, or *Paracoccidiodes* and *Cryptococcus*), and algal (*Prototheca*) cells [10,68,94]. For the TSA-treated cells, loss of ergosterol did not correspond to the overall sterol content. Quite unexpectedly, the cellular sterol content rather than decreasing markedly, increased to more than the control cells. Here, the sterol profile typically was framed around the accumulation of intermediate that served as the substrate for the C24-SMT, which we determined in *Candida* is zymosterol, in *Paracoccidiodes* and *Cryptococcus* is lanosterol while in *Prototheca* is cycloartenol [10,68,94]. The increases in sterol mass reflect presumed increases in newly synthesized C24-SMT enzyme brought about by genetic upregulation in protein synthesis due to the treatment. We have observed this to be true for *C. neoformans* using C24-SMT antibodies to quantify the protein (Nes, W.D., unpublished). Although these TSA treatments can promote growth inhibition at very low IC_50_ concentrations, i.e., in the low nanomolar range in most cases, the inhibitor is typically fungistatic most likely from the reserve sterol pool accumulated in the treated cell which can provide new ergosterol once the treated cells are placed into fresh medium devoid of TSA.

When TSA inhibitors are incubated with trypanosomes the IC_50_ values in cell-based studies are typically in the low micromolar range and the total sterol content is about two-thirds that of control cells [95]. Similarly, *Acanthamoeba* cells treated with TSA produce about half the sterol produced in control cells with a corresponding loss in ergosterol [6,72]. However, in treated cells, there was not much accumulation of sterol biosynthesis intermediates. Together, these studies suggest that TSA treatment of protozoa is not associated with a compensatory change in total sterol content as it appears so in fungi treated with TSA.

So far, we have been discussing synthetic TSA influences on ergosterol-dependency. Antifungal sterol-like compounds (steroidal alkaloids) and their structural congeners are examples of natural products that have been shown to generate the same end response toward ergosterol-dependency. Solasodine and solanidine from the potato family, possess a Δ^5^-monene in the ring and nitrogen atom in the fifth ring or modified cyclized side chain system. Structurally, the steroidal alkaloids can mimic a TSA and inhibit C24-sterol methylation activity in vitro (*K_i_* in low nanomolar range) and can inhibit ergosterol biosynthesis and growth (IC_50_ in the low nanomolar range [69,96]. These compounds, although potent as antifungals [96], proved untenable as therapeutic leads because they were found to be highly toxic to humans [97]. Another approach to identify new sterol methylation inhibitors, that might involve TSA compounds, is through molecular modelling efforts against C24-SMT [98]. However, the current effectiveness of molecular modeling without the background knowledge of a crystal structure which among other things give a precise determination of active site volume and contact residues for the different C24-SMTs and in failing to recognize the native subunit organization of the C24-SMT enzyme, which implies two binding sites for SAM in C24-SMT models, likely will provide a narrow window of reliable drug leads, if at all.

### 3.2. “Bait and Switch” to Irreversibly Inhibit Ergosterol Synthesis in Parasites, a New Concept

Thus far, the pharmaceutical field has focused primarily on azole synthetics as antifungals, and to a lesser extent, as repurposed anti-trypanosomals. Although the structure of these compounds is unrelated to the structure of the sterol substrate, they are effective reversible inhibitors due to their mechanism-based design focusing on the azole nitrogen that bound in the active site can prevent substrate binding and disrupt sterol C14-demethylation catalysis. Our approach was different from the phenotypic screening efforts applied to azole inhibitors—we chose to rationally design, prepare, and test a series of substrate analogs to act as time-dependent inhibitors [99,100,101,102] of C24-SMT or C14-SDM. As reported [72,99,100,101,103] we would evaluate kinetically their inhibitor properties against the cell-free enzyme, and if potent, determine whether they could deplete parasitic cells of ergosterol and in doing so prevent growth irreversibly. For these studies, we developed several therapeutic leads through a process we now refer to as a “Bait and Switch” technique. Here, the natural substrate of the enzymatic bait—C24-SMT or C14-SDM—can be switched with a substrate mimic-antimetabolite bearing a warhead that can explode upon catalysis resulting in covalent binding of the aberrant HEI to these enzymes, and when in vivo, can lead to disruption of ergosterol production in parasites. Because these substrate mimics are in effect structural analogs of cholesterol, we further reasoned they should be acceptable to bind lipoproteins and metabolize in similar fashion to cholesterol by liver enzymes, and therefore not harm the treated human host.

**Figure 6:**
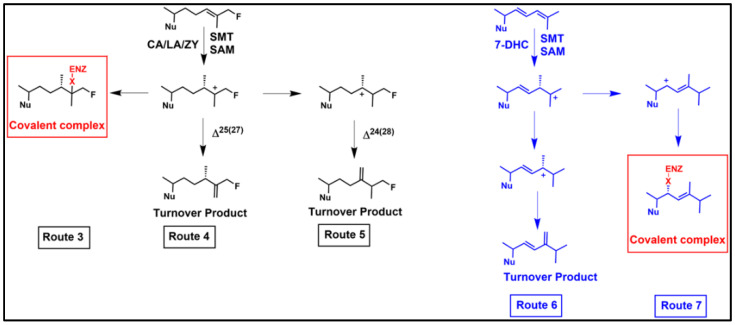
Proposed sterol methylation outcomes for synthetic-C26-F sterol derivative and natural product cholesta-5,7,22,24-tetraenol (CHT) with a C24-SMT; Enz, Enzyme; Nu, sterol nucleus; 7-DHC, 7-dehydrocholesterol or cholesta-5,7,-dienol.

**Figure 7:**
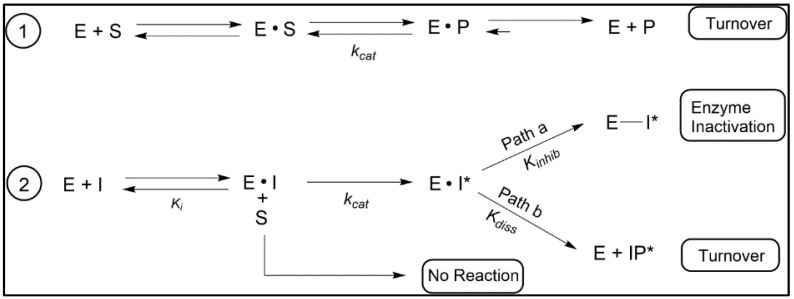
Kinetic schemes for C24-SMT and C14-SDM catalysis. The first step proceeds with physical binding of ligand yielding an E (enzyme)·S (substrate) or E· I (enzyme-inhibitor) complex that is followed by a chemical step (*k_cat_*) involving conversion of substrate (S) to product (P). The initial enzymic step is reversible and considered noncovalent binding of S/I to enzyme. Second in reaction sequence is the chemical step that provides alternate products and other outcomes: Path a proceeds for an irreversible inhibitor such that generation of sterol methylation (C24-SMT) or sterol oxidation (C14-SDM) yields an activated complex (E · I*) that proceeds to an intermediate ion that can be active site quenched leading to enzyme inactivation or for Path b the intermediate ion can be stabilized via cation-ion interaction that then proceeds along a phyla-specific reaction trajectory to product (=turnover) that dissociate the enzyme complex (E + IP*) and enter the sterol biosynthetic pathway as anti-ergosterol surrogates; adapted from [72,99,100].

The suicide substrate features developed for inhibition of C24-SMT require much the same components as required of the native substrate for sterol methylation [70], including a C3-OH, planar nucleus and intact side chain of 8-carbons and an olefinic bond ([86] and references cited therein). However, the functional group subject to sterol methylation can, in contrast to the native substrate, yield one of two outcomes of turnover or covalent attachment depending on the orientation of the bound side chain. The proportion of turnover versus covalent binding ultimately determines the potency of the suicide substrates as a suicide inhibitor. The ability to distinguish between a reversible-and irreversible-type enzyme inhibitor is best represented by two competing processes of partitioning substrate/inhibitor between initial binding and release versus turnover and inactivation/covalent complex (Figure 6 and Figure 7). Criteria implicit in this model, which we have presented in our work on inhibition of C24-SMTs in non-pathogenic and pathogenic organisms involves: (1) proportionality between product and inactive analog-enzyme complex, (2) time-dependent loss of enzyme activity, (3) competitive type inhibition and saturation kinetics, (4) irreversibility established by tandem mass spectroscopy of inhibitor-protein complex, (5) stoichiometric proportionality between covalent modification and enzyme activity, and (6) chemical evidence of the substrate analog bound to the enzyme by, for example for sterol, saponifying the complex and identify the resulting diol (assuming ester bond forms between sterol and enzyme) product by mass spectroscopy and proton nuclear magnetic resonance spectroscopy [72,86,87,101]. However, issues with enzyme preparation of membrane-bound steroidogenesis enzymes and relevant drug lipophilicity that target C14-SDM (CYP51) inhibition have limited the use of time-dependent inhibition studies for kinetic proof of irreversible inhibitor behavior [103].

Kinetic assays using an initial rate and enzyme activity lost following 1 h incubation for time-dependent inactivation of enzyme is the best current approach for the identification and confirmation of the irreversible nature of azole or sterol analog binding to CYP51 enzymes. To establish the inhibitor potency of covalent binding of inhibitor in the presence or absence of native substrate, four independent kinetic equations are often used, one of which recognizes the chemical step (*k_cat_*; Figure 7). Thus, they are: the specificity constant (*k_cat_*/*K_m_*), inhibitor constant (*K_m_*/*K_i_*) where the larger the value the greater the competitive inhibition, a covalent efficiency constant or partition ratio (*k_inact_*/*K_i_*) which can also be expressed as the catalytic constant of the analog—*k_cat_* relative to the time-dependent inactivation constant-*k_inact_*—*k_cat_*/*k_inact_* where the higher the value the more substrate is converted to product than covalently bound to the enzyme.

When the native substrate and analog inhibitor are tested together, and the native substrate *K_m_* differs three or more orders of magnitude more than the analog *K_i_*—the inhibitor is usually considered a transition state analog (TSA), as often seen in studies of C24-SMT. On the other hand, when the native substrate *K_m_* and analog *K_i_* are about the same order of magnitude the inhibitor is generally viewed as in competition with the substrate for binding efficiency and could be a dead-end analog or suicide substrate. If the enzyme is inactivated by the suicide substrate [80,86], as predicted through the formation of an ester bond with the sterol, then the attached ligand can be released by saponification and the resulting product characterized by GC-MS and such “diol” products (one hydroxyl group will be at C3, while the other hydroxyl group will be in the sterol side chain corresponding to where the intermediate cation was produced during catalysis) are assumed to reflect the bound intermediate. In our chemical analyses, we have determined for specific analogs such as those with a methylenecyclopropyl containing side chain that the partition ratio of turnover to covalent bound formation can be expressed mathematically in the form of a C3-monol product (native turnover product) relative to C24/C27-diol product (covalently bound product), as noted in Figure 5 [86,87] or as the case may be for C26-fluorinated inhibitors the partition ratio can be related to C3-monol native turnover product to C3-monol/C26-fluorinated product [100,101].

### 3.3. Aberrant Electrophiles and Strategies for Irreversible-Type C24-SMT Inhibitors as Therapeutic Leads

Given the early success of mechanism-based and targeted irreversible inhibitors resulting in a range of broad-spectrum covalent drugs [102], including the fluorinated drug Vaniqa that was designed for a specific polyamine biosynthetic enzyme (ornithine decarboxylase) crucial in growth of *T. brucei* [104], and the realization that TSA analogs were not viable as antifungal or anti-protozoan drug leads, we began to look into alternative approaches for the design of ergosterol biosynthesis inhibitors that could covalently inactivate C24-SMT enzymes. Our first efforts focused on fungal systems with the target of Erg6p, mainly because we had its substrate and kinetic properties thoroughly characterized. Considering the C24-sterol methylation reaction for C24(28)-methylene product outcome and zymosterol as the basis for substrate analog design, we prepared 26,27-dehydrozymosterol (DHZ) along with 26,27-dehydrolanosterol (DHL) proposing that a sterol side chain cyclopropyl ring can be reactive undergoing metabolism during sterol methylation to multiple products [86]. According to our plan DHZ/DHL could bind and convert to an intermediate with cations at C24 and C27 that either turnover to a C26-methyl product with an elongated sterol side chain and/or covalently attaches to the C24-SMT during catalysis through interaction with one or two of these cations [86] (Figure 5, Route 2).

Indeed, consistent with the enzyme substrate preferences, DHZ was found to bind to the enzyme and showed time-dependent inactivation kinetics against Erg6p and competitive-type inhibitor kinetics versus zymosterol yielding a partition ratio of *k_inact_* 1.52 min^−1^/*K_i_* 48 µM [86,87]. When the inhibitor-enzyme complex was purified by FPLC of the native Erg6p and Erg6-bound by DHZ (confirmed by Tandem-MS and saponification of the enzyme complex and GCMS analysis of extract showing monol and diol products) a start observation revealed the native enzyme eluted a few minutes longer (more polar) than the complexed-enzyme (less polar), consistent with the enzyme having undergone conformational changes during the activation process [82]. In related unpublished studies from the Nes laboratory on cloned TbSMT and cloned AcSMT1, we have observed similar chromatographic differences between the native C24-SMT and one that is complexed with DHZ or DHL, respectively, which supports the view that protein structural changes accompany the irreversible binding of the suicide inhibitor. In further structure-activity studies on Erg6p, 26,27-dehydrolanosterol (DHL) failed to bind productively, in agreement with the substrate specificity of the yeast enzyme. DHZ tested with the *C. albicans* C24-SMT which prefers zymosterol as substrate afforded a partition ratio of *k_inact_* of 0.03 min^−1^/*K_i_* of 9 µM [81] while DHL tested against the *Paracoccidiodes* C24-SMT which prefers lanosterol afforded a partition ratio of *k_inact_* of 0.24 min^−1^/*K_i_* 54 µM [94]. These findings against fungal C24-SMTs of varied substrate specificities and suicide substrate sensitivities reveal distinct differences in the active sites of these enzymes in terms of their amino acid compositions that affect the steric-electric interactions with bound inhibitor and covalency of the reaction. When DHZ was incubated with various fungi, it failed to accumulate in the cells, which was to be expected as yeast typically cannot take up dietary sterols under aerobic conditions [105] and they have a cell wall. Therefore, we stopped studying fungi as an in vivo system to evaluate the steroid-based suicide substrates and considered other pathogenic organisms deemed ergosterol-dependent.

There had been much study on TSA inhibitors against trypanosomes prior to our involvement [106,107], but nothing to do with suicide inhibitors targeting ergosterol biosynthesis. Because trypanosomes and amoebae parasites lack a cell wall and can effectively accumulate large amounts of cholesterol from the growth medium depending on the cell-type or host blood, which we confirmed [14,15,95], we turned our attention to these protozoa for evaluating suicide inhibitors in vivo and against the cell-free C24-SMT. It was understood that the substrate analogs must not only be actively accumulated by cells but they must outcompete the endogenous sterol substrate (biosynthetic intermediate) for binding C24-SMT enzymes [14,16,100,101]. In other cases where sterols can accumulate in sterol auxotrophs—pathogenic fungal cells [108] or intact nematodes [109], the difference between them and the ergosterol synthesizing protozoa stems from the fact that these organisms are auxotrophic for sterols for growth.

To continue with structure-function analyses of suicide substrates we required ample compound for large growth studies to include sterol analysis of treated cells. Unfortunately, zymosterol, the preferred substrate of TbSMT-zymosterol, is not available in commercial quantities for synthetic modification but lanosterol can be obtained from commercial lanolin. Given the limited availability of zymosterol analog to test in cell-based cultures, we prepared from lanosterol both the DHL [100] and a C26-fluorolanosterol derivative [99] because the fluorine atom being highly electronegative should slow the transmethylation reaction—thus enabling alternate sterol methylation trajectories [101] to be exploited in cell-based trypanosome or amoeba cultures. Both lanosterol analogs tested against *T. brucei* were shown to inhibit growth with EC_50_ values in the low μM range while having no effect on human embryonic kidney (HEK) cells to 100 μM, yielding a selective index (EC_50_ HEK cells/EC_50_ *T. brucei* cells) <10 [99,100]. The treated cells at minimum inhibitory concentrations were microscopically dead and could not regenerate when placed into fresh medium, which contrasts with the effects of TSA inhibitors on fungal growth. Another contrasting feature originates in the sterol profiles of DHL and C26-flurolanosterol treated cells. Lanosterol-based TSA inhibitors can accumulate into cells without further metabolism and cause an accumulation of membrane- harmful C4-C14-methyl sterols thereby generating a loss of ergosterol. When lanosterol-based suicide inhibitors (DHL or C26-fluorolanosterol) were supplied to trypanosome cell cultures metabolic studies, it revealed that the inhibitor entered cells and underwent partial conversion by C24-SMT to final C24-methyl Δ^25(27)^-olefinic products not further metabolized. Together, the analog induced-TbSMT killing and alternate C24-methyl product from suicide inhibitor sterol methylation disallowed leakiness in ergosterol synthesis which provided the resulting ergosterol-depletion in treated cells [99,100]. These observations eliminated the possibility for lanosterol derivatives uptake and metabolism by cells and showed for the first time, examples of steroidal pro-drugs as anti-parasitic agents. It is important to note that the addition of a fluorine atom per se to the sterol structure has in the past been considered inert without effect in fungi on ergosterol-controlled growth [110] but when attached to C4 of the sterol nucleus it can interrupt the newly discovered C4-sterol methylation and disrupt larva sterol-controlled growth in nematodes [109]. Alternatively, the addition of a polar group in the form of an oxo or hydroxyl group in the terminal segment of the sterol side chain is reportedly not inhibitory to C24-SMT activities [111].

Chemical and kinetic model studies using zymosterol and the C26F-sterol analog generated metabolically from the C26F-pro-drug supplied to *T. brucei* demonstrated that the fluorinated substrate mimic was a competitive inhibitor and that it was converted by the enzyme to C24-methyl Δ^25(27)^-olefinic product slower than the natural substrate-*k_cat_* 0.26 min^−1^ versus *k_cat_* 0.6 min^−1^, respectively [99]. The C26F-substrate analog can also convert through an alternative sterol methylation trajectory to complex covalently with enzyme (confirmed by time-dependent experiments yielding *k_inact_* 0.24 min^−1^ and GCMS analysis of the monol-diol profile of the enzyme reacted extract), showing another side chain orientation bound to the active site (viz. a second SMT conformation) enabling contact with otherwise cryptic residues that interact with the intermediate ion and inactivate the reaction course. In similar fashion, when the C26F-cycloartenol derivative was tested against the plant (soybean) C24-SMT enzyme in vitro, and kinetically showed covalent binding (*k_inact_* of 26F-cycloartenol 0.12 min^−1^) and GC-MS analysis of the enzyme treated preparations showed multiple C26F-turnover products, one of which the slow- forming product-Δ^25(27)^-olefin was an uncommon 24-methyl sterol for soybean GmSMT1 catalysis (Figure 6, route 4) [101]. These data confirmed the proposal [61] that two primary C24-methyl HEIs in the transition state coordinate occur during the sterol methylation reaction prior to or concomitant with deprotonation of C27 or C28 in the sterol side chain, and as such, should be considered in suicide inhibitor design. Further confirmation for the proposed HEIs formed during sterol methylation incorporates critical information from TSA inhibition characteristics of inhibited plant C24-SMT, and site-directed mutagenesis and affinity labeling experiments on Erg6p against zymosterol and DHZ. The combination of results support the catalytic role of HEI-C24 and HEI-C25 in catalysis and the concerted reaction for sterol methylation [70], and in the catalytic site places glutamic acid flanking Region I as an essential active site amino acid which in Erg6p can contribute to the chemical step by quenching an aberrant intermediate cation to form an ester bond yielding covalent complex of the bound substrate rather than enabling the intermediate cation to proceed with deprotonation for turnover [29,75,82,86].

In other routine screening of steroidal analogs common to yeast used as negative controls for growth inhibition, we observed quite unexpectedly that cholesta-5,7,22,24-tetraenol (CHT) and ergosta-5,7,22,24(28)-tetraenol (ERGT), generated rapid cell death against the parasitic *A. castellanii* and *T. brucei* cells [112,113]. Since there was no precedent for the induced toxicity in protozoa or any other eukaryotic system resulting from feeding yeast sterols to the growth media, the growth responses were anomalous. The bloodstream form of *T. brucei* cultures incubated with CHT and ERGT displayed EC_50_ values of 2.9 nM and 52 nM, respectively, with total cell death from CHT occurring within hours of cell treatment. In similar fashion, CHT and ERGT independently were shown to inhibit growth of *A. castellanii* trophozoites generating IC_50_ values of 51 nM with total cell lysis occurring within 48 h of treatment. Further, testing CHT in a mouse model of *T. brucei* infection led to significant increases in survival time following daily treatment for 8–10 days at 50 mg/kg or 100 mg/kg [113], showing promise for these compounds as therapeutic leads. Incubation of CHT and ERGT up to 40 μM with HEK cells failed to affect growth, even though the compounds were accumulated by the cells, and minimally metabolized in the ring system of Δ^5,7^ to Δ^5^ (as in cholesterol biosynthesis)—not side chain, and they did not have any effect on cholesterol production. The selective index (human/treated parasite cells) is <5000, exhibiting a great deal of drug potency.

The mode of inhibitor action of the novel growth inhibitions by yeast sterols against two protozoa from different evolutionary lineages became evident from sterol analysis of the treated cells which showed ergosterol synthesis blocked and altered the sterol profile to relate to inhibited C24-SMT activity. Consequently, we next focused our investigations on incubations of CHT and ERGT with cloned C24-SMTs from *T. brucei* and *A. castellani*. Kinetic study of bound yeast sterols to TbSMT1, AcSMT1 and AcSMT2, chemical analysis of the analog covalently bound to C24-SMT by Tandem MS, mutagenesis in Region I of the C24-SMT, and GCMS analysis of extracts of enzymes reacted with the yeast sterols showed that the active site of this enzyme from different protozoa recognized the yeast sterols [72,112,113]. What made the natural product yeast sterols even more potent than the synthetic analogs we had designed, based on their structural attributes, to generate HEIs at C24 and C25, was the apparent misplacement of the sterol side chain in a region of the active site not normally occupied by the intermediate ion. This therefore led to C22-cation formation and the resulting covalent complex. Interestingly, when comparing the suicide inhibitor sterol methylation routes to protein alkylation as shown in Figure 6, Route 7 compared to Route 6, CHT (and ERGT as well) enzyme features possessing the Δ^25(27)^-route were much more sensitive to the conjugated Δ^22,24^ diene sterol side chain than for example Erg6p which can convert CHT to ERGT without alternative outcomes. In vivo, the protozoan SMTs that operate the ∆^25(27)^-route are more sensitive to irreversible knock out by ∆^22,24^-steroidal antimetabolites compared to organisms and C24-SMTs that operate the ∆^24(28)^-routes. Thus, Δ^22,24^-sterol side chain scaffolds generating the novel antimetabolite qualities observed in CHT and ERGT are notable and warrant further study against parasites synthesizing the Δ^25(27)^-olefins, particularly focusing on changes in sterol profiles and the mRNA levels and rates of C24-SMT and C14-SDM enzyme activities following drug treatment.

## 4. C14-SDM Sterol Biosynthesis Inhibitors

### 4.1. C14-SDM Catalytic Competence and Properties

**Figure 8:**
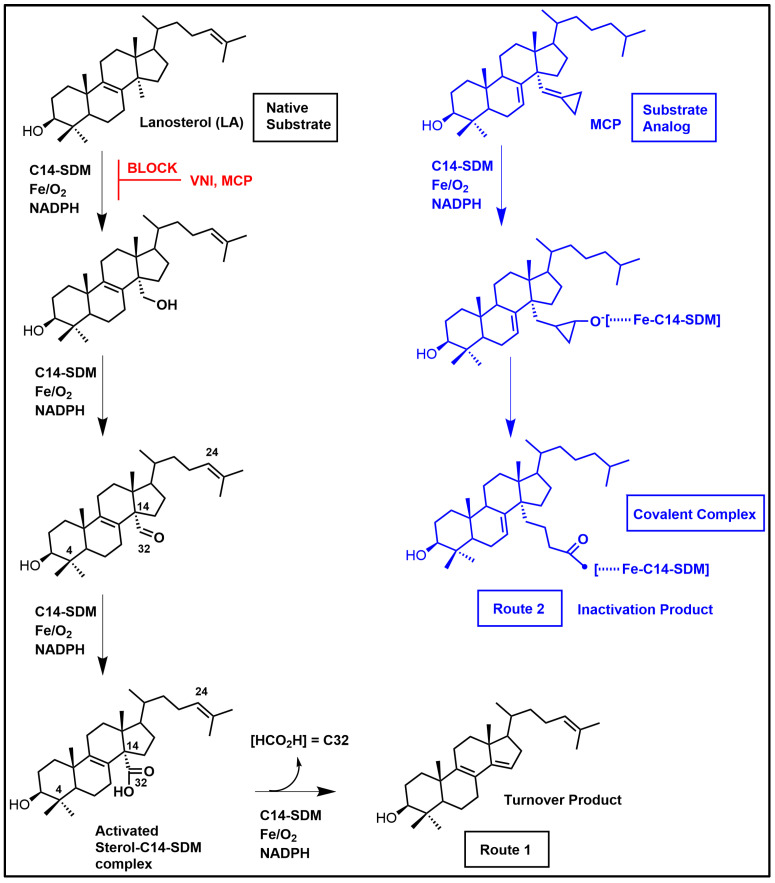
Lanosterol C14-demethylation pathway to substrate turnover (Route 1) and proposed blockage to substrate turnover by a MCP-suicide inhibitor complex (Route 2).

Sterol C14α-demethylase (C14-SDM = CYP51 = Erg11p), a sterolic enzyme that is central to ergosterol biosynthesis in pathogenic fungi and protozoa and in cholesterol biosynthesis in the human host, has received much attention for its targeting in ergosterol synthesis. C14-SDM catalyzes the stepwise oxidative removal of the C-14 group (viz. C32) from lanosterol with the aid of cofactors molecular oxygen, Fe and NADPH (Figure 8) [1,6,114]. The heme cofactor is tethered to the proximal side of the protein via a thiolate ligand derived from a cysteine residue that serves an electron donor in C14-demethylase catalysis. The eukaryotic enzyme is believed to have originated in bacteria [115] where the CYP51 enzyme has specific substrate structure requirements that do not catalytically tolerate the plant protosterol, cycloartenol (Figure 3) [42]. Typically, cycloartenol is understood to convert (via 9,19-cyclopropane ring opening) to a ∆^8^-sterol, that becomes the substrate for CYP51 enzymes in these systems and the products of CYP51 catalysis proceed to Δ^5^-sterols (Figure 1).

Cycloartenol, a structural isomer of lanosterol, is now known to be conformationally flat—not bent—as once considered in substrate binding studies and in plant-animal steroidogenesis experiments [31,116]. For CYP51 catalysis, the substrate is assumed to possess a Δ^8^ or Δ^7^-bond in the sterol nucleus and requires an active proton delivery network that supports a compound-I-mediated C-C bond cleavage in the reaction sequence [6,117]. That the eukaryote CYP51 substrate preference could be broader than previously realized was underscored by recent structure-activity studies on AcCYP51 that involved the typical plant sterol-cycloeucalenol (31-nor 24(28)-methylene cycloartenol). Here, catalysis generated a single product of cyclopropyl containing sterol bearing a Δ^14(15)^-bond. The product identity was confirmed by GCMS analysis which can detect catalytic outcome at levels smaller than those obtained by simple kinetic experiments [14]. Clearly, more work on a range of CYP51 targets incubated with various cyclopropyl containing sterols is warranted to adequately address the structural requirements of substrate for CYP51 catalysis in parasitic protozoa.

**Figure 9:**
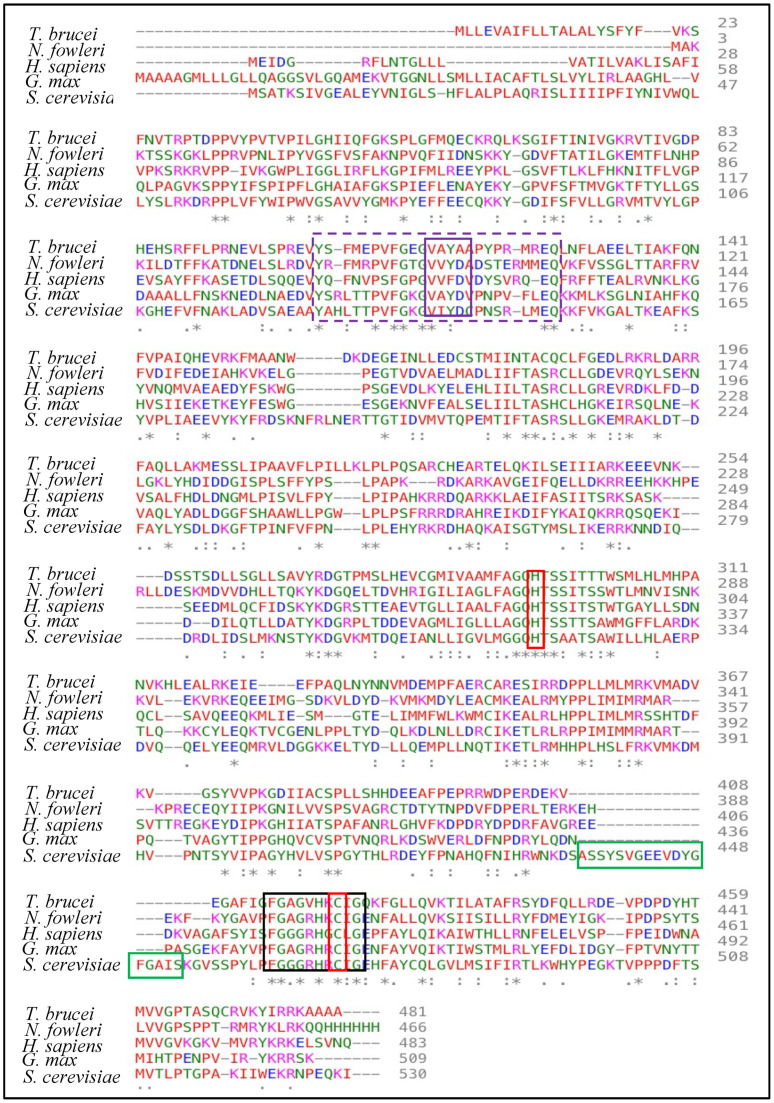
Primary sequences for the sterol-14-alpha demethylase from *T. brucei* (Q385E8) (54.4 kDa), *N. fowleri* (A0A2H4A2U9) (53.5 kDa), *H. sapiens* (Q16850) (57.3 kDa), *G. max* (K7KEG4) (54.8 kDa), *and S. cerevisiae* (P10614) (60.7 kDa) were aligned using Clustal Omega. The heme-coordinated cysteine and the conserved CYP51-specific histidine are in red boxes, while fungal-specific sequences are in green boxes. The major sterol substrate-binding region is in a box with purple dashed line. Cyp51 signature sequences are shown in boxes with black and purple solid lines. Acidic, basic, hydrophobic, and polar residues are in blue, pink, red, and green letters, respectively. * Indicates conserved residues, : indicates stronger similarity regions, and . indicates weaker similarity section.

The primary amino acid sequences for a range of C14-SDM enzymes are shown in Figure 9 and the conserved heme-coordinated cysteine, the conserved C14-SDM-specific histidine, the major substrate-binding region in addition to C14-SDM signature sequences are readily identifiable. According to X-ray structures of this class of enzyme, the proximal face of eukaryotic C14-SDMs exhibits a considerable increase in positive electric charge that complements electron transfer partners and this increase in positive charge is noticeable across eukaryotic lineages [114]. As to be expected, the interior cleft is reasonably hydrophobic to accept greasy steroidal substrates (and mimics).

The native molecular weight of human C14-SDM is 57.3 kDa and it crystallizes as a homodimer [118,119]. The conserved C14-SDM heme coordinated cysteine and histidine residues across Kingdoms have been identified in Figure 9 in addition to the conserved major C14-SDM substrate-binding region, C14-SDM signature sequences and fungal-specific C14-SDM sequences. Several cloned C14-SDM enzymes from plants/trypanosomes and fungi/animals have been characterized for substrate specificity and reaction rate.

Thus, the former group prefers C4-mono methyl sterol substrates while the latter group prefers C4-dimethyl sterol substrates (Figure 3) with reaction rates associated with these substrates slower in the former group (ca., 5 min^−1^) and faster in the latter group (ca., 30 min^−1^) [37]. The differences in substrate recognition can be explained in part by the protein structure. Analysis of variant CYP51 amino acid sequences reveals that those of trypanosome have less than 25% identity with their fungal orthologs. Recent structure determinations correlated to activity assay of substrate analog and azole binding to wild-type C14-SDM enzymes, and their corresponding mutants revealed key active site residues in the substrate binding segments of plant/trypanosomes versus fungus/human CYP51s provide structural rigidity of the CYP51 binding cavity involved with the enzyme ability to preserve strict catalytic activity and give way to changed substrate requirements in a phyla-specific manner [38,87,118].

While conventional wisdom assumed that sterol biosynthesis from protosterol to Δ^5^-sterol involved a single set of genes coding for individual sterolic enzymes (one-gene/one-enzyme theory) in the pathway, more recent molecular biology of steroidogenesis details tell a different story. Thus, yeast, human and other vertebrate have one CYP51 gene (or C14-SDM), but other fungi, like *A. fumigatus* have two paralogs, CYP51A and CYP51B. The later gene encodes the enzyme primarily responsible for sterol C14-demethylation and it is expressed constitutively in all sequenced filamentous fungus. Whereas CYP51A gene appears in some fungal lineages [120,121,122]. Plants also have multiple copies of CYP51 genes, but some may be redundant [123]. Similarly, plants often possess a family of SMT1 and SMT2 enzymes where individual member enzymes are functionally distinct, catalyzing specific sterol methylation steps that govern the overall 24-alkyl phytosterol production, and one of SMT2-1 and SMT2-2 isoforms is seemingly catalytically redundant [79].

### 4.2. Phenotypic Screening of Azoles—Basis for the Druggability of Protozoan CYP51 Enzymes

**Figure 10:**
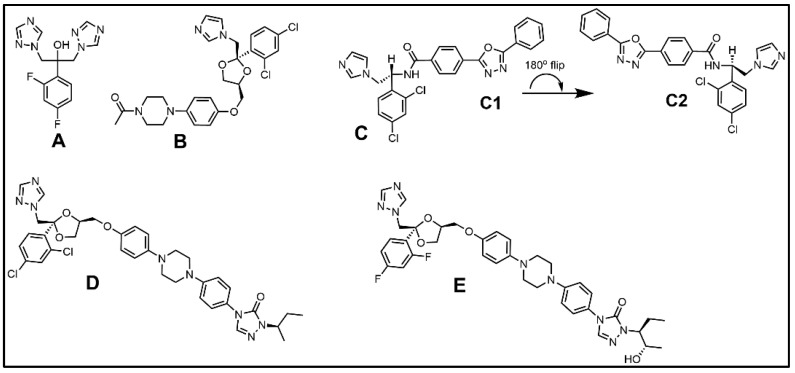
Representative azole-type ergosterol biosynthesis inhibitors: (**A**) Fluconazole, (**B**) Ketoconazole, (**C**) VNI, (**D**) Itraconazole, and (**E**) Posaconazole. Note: VNI can exist in alternate conformations (**C1**,**C2**). (**C2**) matches the drug orientation aligned with TbCYP51 contact residues associated with bound MCP C3-OH and C14-methyl groups as discussed in text.

The therapeutically relevant azole-containing antifungal compounds selected for phenotypic screening in diverse parasitic protozoa have long been recognized as possibilities for repurposed drugs to target the CYP51 enzyme in infectious trypanosomes and amoeba e.g., ketoconazole (Figure 10B) [124,125,126]. In the antifungal market, ketoconazole came first as an effective derivative of miconazole, followed by its 1,2,4-triazole analog itraconazole, then posaconazole an itraconazole derivative, all which showed varying antifungal effectiveness for systemic use [127]. Fluconazole is another water soluble triazole derivative of miconazole developed for inhibiting ergosterol biosynthesis but rather than being a broad-spectrum antifungal, fluconazole is limited to treating pathogenic yeast (Figure 10). However, by altering the core structure where two of the triazole rings are replaced with the fluoropyrimidine ring the resulting voriconazole generated improved antifungal activity across a greater number of pathogenic fungi [127]. As predicted from studies of azole-treated fungi, the ketoconazole-treated trypanosomes generated an increase in C14-methyl sterols and loss of ergosterol [124], consistent with the drug target CYP51 having undergone blockage by the drug. Early on, some azoles were shown to be generating curative, rather than suppressive activity against *T. cruzi* causing Chagas disease [126]. More recently, novel azole structures-VNI (Figure 10) have provided even greater potency and curative effectiveness, and it is noted that VNI and poscaconazole eliminated the parasite in experimental models of acute and chronic Chagas disease [128], giving further excitement to the possibility of developing them as anti-parasitic agents broadly. Yet, caution should be exercised since *T. cruzi* cells incubated with azoles, other than VNI, may produce an upregulation in mRNA associated with CYP51 protein potentially generating an abundance of new enzyme causing leakiness in the ergosterol biosynthesis pathway [129], as noted before for treated fungi.

When the conazole drugs, fluconazole, itraconazole, voriconazole and posaconazole, discussed above were investigated more recently against the parasitic *Acanthamoeba* and *Naegleria* amoebae, the in vivo and in vitro results validated CYP51 as a potentially “druggable” target for the treatment of keratitis and granulomatous amoebic encephalitis, and primary amoebic meningoencephalitis (Table 1) [130,131]. Perhaps the most significant finding of the drugs tested is that fluconazole binds weakly to AcCYP51 generating a dissociation constant (*K_d_* = 2.1 μM) and did not inhibit cell growth to >64 mg/L, while voriconazole binds tightly (*K_d_* = 13 nM) and kills cells at a minimum inhibitor concentration of 1–2 mg/L [131].

### 4.3. CYP51 Inhibitors: First Efforts for Sterol-Based Substrate Analogs in Treating Heart Disease

With the cholesterol biosynthesis pathway elucidated, with the enzyme specificities of the steroidogenesis enzymes determined, and heart disease on the rise—inhibition of lanosterol metabolism in cholesterol biosynthesis became a focus of rational design in substrate analog investigation in the 1980s and 1990s that included the introduction of fluorine atoms and nitrogen atoms at C15 in the sterol nucleus [132,133,134]. Lanosterol-based substrate analogs equipped with a fluorine warhead became the first generation of steroidal suicide inhibitors designed to disrupt cholesterol synthesis with the plan to patent effective analogs as heart drugs. Mechanistically, the modified substrate analog was proposed to interfere with the C14-demethylation process to generate oxygenated intermediates on C32 that could feedback and down-regulate HMG-CoA reductase, the rate-limiting enzyme in cholesterol synthesis. Extended cell-based studies were carried forth by industrial researchers on C15F-lanosterol supplied to hepatocyte cultures establishing the accumulation of C15F-lanosterol oxygenated metabolites and shut down of HMG-CoA-reductase activity [48,49,134]. When the C15F- sterol was examined against a C14-SDM enzyme preparation, the analog was shown to convert to an oxygenated C15F, C32 al-derivative [48]. The fluorine atom introduced at C15 played the same mechanistic role observed for the fluorine introduced at C26, in slowing enzymatic reaction enabling reaction intermediates to accumulate. Unclear in these industry studies was whether the C15-lanosterol analog underwent metabolism by C14-SDM to bind covalently. Because of the rise in blockbuster statins against HMG-CoA reductase to successfully treat heart disease, industrial interest in this line of research targeting human cholesterol biosynthesis ceased.

### 4.4. CYP51 Inhibitors: Sterol-Based Analogs for Treating Parasitic Disease

By the mid-1970s, a new class of enzyme inhibitors often referred to as mechanism- based *k_cat_* inhibitors (Section 3.2) were evolving as potential therapeutic agents, but none of them were considered against steroidogenesis in parasitic protozoa [135,136,137]. The first compound to pioneer the development of substrate analogs as antimetabolite or suicide substrate targeting C14-SDM was designed and prepared in the Nes laboratory—14α-methylenecyclopropyl-Δ^7^-24,25-dihydrolanaosterol (MCP) [103]. This substrate analog was provided to our colleagues at Vanderbilt University and Meharry Medical College, and they tested it against trypanosome cell cultures and TbCYP51, which included kinetic and structure determinations of the enzyme complexed with MCP. The potency of the novel steroidal analog bearing a methylene cyclopropane group tested against C14-SDM, like that of the sterol side chain methylenecyclopropyl-containing DHZ and DHL tested against C24-SMT, generated binding constants of approximately 0.5 μM [87,103], showing an acceptable affinity for the enzyme. In cell-based *T. brucei* and *T. cruzi*, MCP was shown to inhibit growth generating IC_50_ of 6 µM and against the cell-free enzyme to exhibit time-dependent inhibition, results that are consistent with its irreversible inhibitor properties [103]. These observations partially satisfied the Bait and Switch approach for *k_cat_* inhibitors specific to blocking ergosterol production in parasites, Section 3.2 [72,100,101,113].

The proposed mechanism of action of MCP considers reaction cycles involved in CYP51 catalysis discussed as follows. The general catalytic scheme for oxidative removal of the C14-methylgroup (=C32) of lanosterol is typically shown as a 3-step process illustrated as in Figure 8 [1,6], while more detailed reaction paths have been reported elsewhere [1,117,132]. One of these mechanisms incorporates transient radical intermediates that can undergo recombination with the iron-bound oxygen atom (anion) in the CYP51 active site ([127,132] and references cited therein). With this in mind, MCP was designed as a suicide substrate to undergo initial oxidation via C14-SDM catalysis, and due to an altered trajectory, that is established upon substrate oxidation—the analog is predicted to covalently bind the enzyme. For MCP covalency, there is a proposed truncated reaction driven by the P450 ferryl porphyrin radical cation intermediate known as compound I, or the ferric hydroperoxide anion that precedes compound I, either of which can react with the methylene cyclopropane generating ring opening to a polar elongated appendage extending from C14 of the sterol nucleus as shown in Figure 8. In support of this proposal, the Waterman group found MCP could complex irreversibly with TbCYP51 and TcCYP51 and established by structure determination, the orientation of this analog in the active site relative to key contact residues and heme [103]. Notably, there have been few crystal structures of sterolic enzymes with a sterol substrate bound in the active site and our work, thanks to the Nashville group, is among the first to show this complex, not only for CYP51, but sterolic enzymes generally.

### 4.5. CYP51 Inhibitors:Azole-Based Time-Dependent Inhibitors for Treating Parasitic Diseases

**Figure 11:**
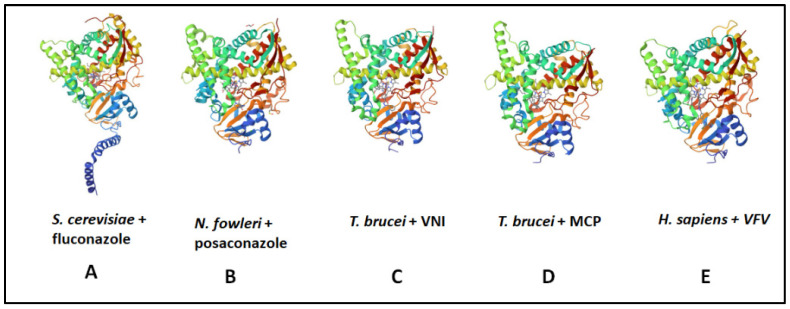
Representative crystal structures of C14-SDM (CYP51) enzymes complexed with reversible structures: (**A**) (PDB: 4WMZ; Ref. [143]) and (**B**) (PDB: 5TL8; Ref. [141]), or irreversible inhibitors: structures (**C**) (PDB: 3GW9; Ref. [138]), (**D**) (PDB: 3P99; Ref. [103]) and (**E**), a fluorinated VNI derivative (PDB: 4UHL; Ref. [118]) as discussed in the text. Crystal structures are presented as ribbon diagrams that are colored as a rainbow spectrum with the N-terminal helix colored blue and the C-terminal helix colored red.

The facility to generate crystal structures of cloned CYP51 enzymes has paved the way for structure-guided investigations of CYP51 enzymes from pathogenic organisms and of CYP51 from humans bound with, for example, the reversible-type inhibitors fluconazole, ketoconazole, itraconazole and posaconazole and irreversible-type inhibitor VNI (Figure 11). Using predictive best-fit modelling of drug-enzyme interactions and reliance on the lipophilicity per hydrogen atom expressed as logP/MW values [138,139,140,141,142] crystallographers have shown relevant differences in the active site of fungal C14-SDM complexed with fluconazole (Figure 11) [143] compared to the trypanosome TbCYP51 complexed with VNI [138]. More specifically, structure determinations coupled to molecular docking of five common antifungal azoles bound to *Naegleria fowlerii* CYP51 (Figure 11) viz. the conazole drugs itraconazole, fluconazole, ketoconazole, voriconazole, and posaconazole, show similarities in the coordination bond to heme iron via aromatic nitrogen of the heterocyclic moiety. Moreover, the overall protein scaffold of NfCYP51 is like that of previously characterized CYP51 from other eukaryotes, yet distinct active site differences exist explaining the relaxed substrate requirements between this enzyme and the TbCYP51 ortholog (Figure 11) [140,141]. Notably, a structurally guided reason explains why the smaller structured fluconazole, excellent as an antifungal, is inferior or physiological incompetent compared to posaconazole in inhibition *Naegleria* growth and ergosterol biosynthesis. Thus, the azole weakly binds the NfCYP51 due to its location in vicinity of the heme where it benefits little from the surrounding active site tunnel space [130]. In contrast, the potent posaconazole in vivo and in vitro occupies the whole length of the NfCYP51 hydrophobic tunnel spanning from the heme macrocycle to the protein surface [131]. It is not surprising then to learn that depending on the drug-CYP51 complex, the azole can orient itself relative to key active site residues (for example, Pro123 in NfCYP51) in different conformations against the different CYP51 enzymes. For example, the long posaconazole shown in Figure 11 can become bent about the piperazine ring at binding to adequately meet the greasy structure criteria for inhibitor action. This conformational adjustment in azole at binding was also found necessary for inhibitor action against TbCYP51 and TcCYP51 [141].

Some uniformity in active site CYP51 structures in pathogenic organisms exists but the nuances in the cleft structure enable bound azole to orient itself relative to key active site residues (for example, Pro123 in NfCYP51) in different conformations for different CYP51s. For example, the long posaconazole shown in Figure 11 can become bent about the piperazine ring at binding to adequately meet the greasy structure criteria for inhibitor action. Conformational adjustment of azoles upon binding is also necessary for inhibitor action against TbCYP51 and TcCYP51 [131]. VNI can exist in alternate projections as shown in Figure 11, C1 and C2, with C2—the conformer that aligns in the TbCYP51 active site positionally equivalent with the face of MCP and its polar C3-OH group in contact with a methionine residue and the tail structure with C14-methyl group buried in a hydrophobic center next to the heme iron [103]. The nuances at the azole binding site that impact in vivo drug potency are also noted in the crystal structures of these enzyme where for example, the trypanosome CYP51 active site impose juxtaposes to produce strict substrate binding in contrast to the amoeba NfCYP51 active site which is more spatially promiscuous to allow for relaxed substrate requirements [131,138,140,141].

Despite numerous azole modifications that have appeared over the years, no azole has been discovered as an irreversible inhibitor through conventional phenotypic screening methods. Alternatively, by monitoring for time-dependent inactivation kinetics in vitro against trypanosome CYP51 enzymes and correlating this in vitro activity to VNI treatments of *T. cruzi* cultured cells, the Nashville group identified VNI as a covalent drug. Furthermore, they identified a novel protozoa-specific inhibitory scaffold in trypanosome CYP51 structures that correlates to the azole part of VNI (Figure 11), not evident in the human CYP51 (Figure 11) [118]. It is through the VNI scaffold (carboxamide containing beta-phenyl imidazoles) that time-dependent inactivation effects can take place against TbCYP51 and TcCYP51 enzymes and from which the killing of trypanosome cells can occur at low nanomolar concentrations [138,141]. Interestingly, the trypanosome growth response to VNI is much more dramatic than MCP by several orders of magnitude, even though both compounds are considered irreversible inhibitors of CYP51 activity. In part, this may be due to a delivery system involved with the accumulation of these compounds into cells or the way the covalency is acquired; more study on this matter is warranted.

## 5. Conclusions and Future Perspectives

What we call the sterol field effectively began with the finding of cholesterol in human gallstones in pre-revolutionary France in the 18th Century (and unearthed again in the early 19th Century) with the foundational discoveries in chemistry and medicine of cholesterol and its structural relatives continuing into the 21st Century. Several internationally renowned scientists have been awarded the Nobel Prize over the last hundred years or received the Schroepfer Medal, initiated in 2002 [6,144,145] for sterol research. Fast forward to today where the sterol field is recognized to involve a multi-disciplinary approach which takes into account: natural product, organic and structural chemistry, molecular biology, and mechanistic enzymology together with traditional biology for cell-based studies and mouse models of infection. One specific area that utilizes all of these approaches involves the rational/structure-guided design and development of ergosterol biosynthesis inhibitors for the treatment of pathogens targeting humans (and crop/ornamental plants) discussed in this review ([39,40,46,146] and references cited therein). Numerous candidate compounds from the current armamentarium of antifungal drugs have been investigated against parasitic protozoa but thus far, most of the antifungal azoles tested fail to adequately treat trypanosome or amoeba under infectious conditions. Another approach is combining different amounts of a TSA and azole against the C24-SMT and C14-SDM enzymes in ergosterol biosynthesis. Hence, one compound could be applied synergistically at trace level versus the other compound at IC_50_ level to off-set any ergosterol leakiness from treatment of the paired inhibitor and it is noted that no such combination has resulted in the advancement of a viable therapeutic combination lead [5,14,15,16,126,127]. An alternative combination therapy is for an antifungal azole coupled to AmB to eliminate ergosterol from cells through different modes of depletion; regrettably, pairing of these compounds failed to translate into clinical success since resistance in the treated organisms was still noticeable [56,147,148].

Happily, there is hope underscored by the recent demonstration of a Bait and Switch approach in suicide inhibitor effectiveness against protozoa C24-SMT and C14-SDM enzymes and in the biochemical novelty of this drug type in generating ergosterol depletion and rapid cell death in parasites. To our credit, together with that of our collaborators at Vanderbilt University, Meharry Medical College, and Swansea University (UK), we demonstrated for the first time in trypanosomes and amoeba systems, the novelty of irreversible inhibitors interfering in ergosterol biosynthesis-one includes VNI in the azole category, and the other includes DHZ, CHT and MCP in the substrate mimic category. It is anticipated new research directed at selectivity and potency of suicide inhibitors should produce novel covalencies in reaction mechanisms affording C24-SMT or C14-SDM inactivation associated with long lifetimes that impact the protein half-life, and in doing so, off-setting any compensatory upregulation in gene expression to produce fresh enzyme to overcome the blocked step. Future work is anticipated to focus on co-metabolite regulation in ergosterol production from inhibition by newly identified suicide substrates and/or azoles, on unrealized strategies for exploiting the target enzymes active site structure (particularly once there is a crystal structure of a C24-SMT), and on the uniqueness of the protozoa-azole scaffolds for design and structure-activity testing of yet unidentified CYP51 inhibitors. The pace of research in these exciting areas will certainly increase as new suicide inhibitors derived synthetically or from natural products, and small molecule library screening efforts are shown to be potent in parasite cell-based cultures and mechanistically shown to exhibit covalent complex leading to sterolic enzyme inactivation. The irreversible inhibitors targeting sterolic enzymes discussed here provide very promising therapeutic leads for mechanism-based covalent drugs as anti-parasitic agents. Indeed, exploiting the mechanistic difference in the development of new, selective *k_cat_* inhibitors against C24-SMT and C14-SDM will provide better treatment profiles for ergosterol-dependent parasites.

## Figures and Tables

**Table 1:** Ergosterol-dependent infectious diseases ^1^.

Disease(s)	Sterol Biosynthetic Features ^2^	Ergosterol Dependency	Infection Site	Representative Infectious Agent	Phylogenetic Lineage
Keratitis and granulomatous amoebic encephalitis	Prototroph: cycloartenol C_28_/C_29_ sterols	Yes	Blood and tissues	*Acanthamoeba* sp.	Amoebozoa
Naegleriasis and primary amoebic encephalitis	Prototroph: cycloartenol C_28_ sterol	Yes	Blood and tissues	*Naegleria* sp.	Heterolobosea
Sleeping sickness	Prototroph: lanosterol C_28_/C_29_ sterols	Yes	Blood and tissues	*Trypanosoma brucei*	Euglenoids
Chagas disease	Prototroph: lanosterol C_28_/C_29_ sterols	Yes	Blood and tissues	*Trypanosoma cruzi*	Euglenoids
Candidiasis	Prototroph: lanosterol C_28_ sterol	Yes	Skin/vagina/ mouth	*Candida albicans*	Holomycota
Cryptococcosis and meningitis	Prototroph: lanosterol C_28_ sterol	Yes	Lungs and brain	*Cryptococcus* sp.	Holomycota
Aspergillosis	Prototroph: lanosterol C_28_/C_29_ sterols	Yes	Lungs	*Aspergillus* sp.	Holomycota
None	Prototroph: lanosterol C_27_ sterol	No	NA	*Homo sapiens*	Holozoa

^1^ References [5,14,15,16,17,18,19,20,21,22]; ^2^ Prototroph terminology is used in context of the organisms ability to biosynthesize sterols; NA = non-applicable.

**Table 2:** Gene-enzyme properties in sterol metabolic pathways across eukaryotic plant-animal kingdoms ^1^.

System(s) ^1^	Function	Common Gene Name			Enzyme Nomenclature	Enzyme Commission (EC) Number	Cofactor
		*A. thaliana*	*S. cerevisiae*	*H. sapiens*			
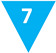	2,3- oxidosqualene cyclization	CAS (7A)	ERG7 (7B)	LSS (7B)	Lanosterol/ Cycloartenol synthase	5.4.99.7/8	NA ^2^
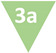	C14-demethylation	CYP51G1	ERG11	CYP51A1	Sterol 14α-methyl demethylase	1.14.13.70	Heme NADPH
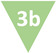	C14-reduction	FK (TM7SF2)	ERG24	DHCR14	Sterol Δ^14^ -reductase	1.3.1.70	NADPH
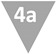	C4-methyl oxidation	SMO1	ERG25	SC4MOL	Sterol C4- methyloxidase	1.14.13.72	Oxo-diiron, NADPH
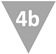	C4-methyl acid elimination	AT3βHSD/ D1	ERG26	NSDHL	3β-Hydroxy- Δ5-steroid dehydrogenase	1.1.1.170	NADH
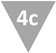	C3- ketoreduction	Predicted ^3^	ERG27	HSD17B7	3β-Keto-reductase	1.1.1.270	NADPH
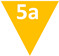	9β, 19β- cyclopropane ring opening	CPI1	NA ^2^	NA ^2^	Cycloeucalenol cycloisomerase	5.5.1.9	NA ^2^
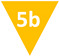	Δ^8^-Δ^7^ isomerization	HYD1	ERG2	EBP	Sterol C8 isomerase	5.3.3.5	NA ^2^
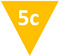	Δ^5^-desaturation	DWF7	ERG3	SC5DL	Sterol C5-desaturase	1.14.19.20	Oxo-diiron, NADPH
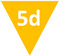	Δ^7^-reduction	DWF5	NA ^2^	DHCR7	Sterol Δ^7^ reductase	1.3.1.21	NADPH
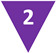	Δ^24^-reduction	DWF1	ERG4	DHCR24	Sterol Δ^24(25)^/Δ^24(28)^ reductase	1.3.1.72	NADPH
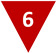	C22- desaturation	CYP710A	ERG5	NA ^2^	Sterol C22 desaturase	1.14.19.41	Heme NADPH
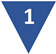	C24/28-methylation	SMT1/2/3	ERG6	NA ^2^	Sterol C24/28-methylase	2.1.1.41/43	SAM

^1^ See Figure 2 for annotations; ^2^ NA = not applicable; ^3^ Gene has not been identified for this enzyme, although it is proven by biochemical/genetic approaches to be in sterol metabolism; Table 2 was adapted from ([1,24,25] and references cited therein). Note: some systems within a triangle can represent multiple enzymatic reactions/steps. For the C24/C28-methylation step, the SMT3 is now known to be a SMT2 isoform and to be functionally redundant, so a better nomenclature we prefer is SMT2-1 and SMT2-2. From unpublished work in the Nes laboratory on phytosterol biosynthesis in *Arabidopsis*, corn, and other plants, it appears there is no Δ^25(27)^-reductase (and no evidence for this enzyme in the gene bank). However, we found the Δ^25(27)^-double bond can isomerize back into the chemically more stable Δ^24(25)^-double bond position for reduction.

**Table 4:** Uniformity and differences in 24-SMT kinetic constants and reaction courses ^1^.

Source	24-SMT Substrate	*K_m_*	*K_cat_*	*E_a_*	Kinetic Mechanism	Reaction Pathway	*M_r_* (kDa)
		(μM)	(min^−1^)	(K_cat_/mol)			
Fungi	*S. cerevisiae* SMT1 ZY	17	0.6	13.7	Random binding Concerted	S_N_2 Δ^24(28)^	40.43
Land Plant	*G. max* SMT1 CA	30	1.3	15.2	Ordered SAM Binds First	S_N_2 Δ^24(28)^	40.40
Land Plant	*G. max* SMT2 ML	26	0.8	21.0	ND ^2^	S_N_2 Δ^24(28)^	42.33
Protozoa	*T. brucei* SMT1 ZY	47	0.6	18.1	ND ^2^	S_N_2 Δ^25(27)^	40.32

^1^ Reported in references [32,41,65,68,72,76,77]. ^2^ Not determined. Substrate: ZY = zymosterol, CA = cycloartenol, ML = 24(28)-methylene lophenol.

## Data Availability

Not applicable.
